# Book Reviews

**Published:** 1897-02

**Authors:** 


### Book Reviews

REFERENCE-BOOK OF PRACTICAL THERAPEUTICS, by various authors. Edited by Frank P. Foster, M. D., Editor of the New York Medical Journal and of Foster's Encyclopœdic Medical Dictionary. In two volumes. Volume I. New York: D. Appleton and Company.

This work constitutes a veritable encyclopœdia of drugs and their application in the treatment of disease. The articles are of length appropriate to the importance of the subject, and are arranged alphabetically—a convenient arrangement for every reference. The topics are signed by the contributors, a desirable feature, which has caused some confusion by being omitted in some of the comprehensive volumes recently issued from another house. The principal contributors are Wyeth, Whittaker, Potter, Leonard, Corning, Gerster, and others. The work is edited by the well-known editor of the *New York Medical Journal*, Dr. Frank P. Foster.

---

A MANUAL OF MATERIA MEDICA AND PHARMACOLOGY. Comprising all Organic and Inorganic Drugs which are and have been official in the United States Pharmacopeia; together with important allied species and useful synthetics. Especially designed for students in pharmacy as well as for druggists. pharmacists and physicians, By David M. R. Culbrith, Ph.G., M. D., Professor of Botany, Materia Medica, and Pharmacology in the Maryland College of Pharmacy. With four hundred and forty-five illustrations. 818 pp. Lea Brothers & Co., Philadelphia and New York. 1896.

This work is recommended to students of pharmacy and materia medica. The illustrations are faithful to nature and will be of great aid to the amateur botanist who wishes to study the resources of his fields and forests.

---

A COMPEND OF GYNECOLOGY by William H. Wells, M. D., Adjunct Professor of Obstetrics and Diseases of Infancy, in the Philadelphia Polyclinic; Late Assistant Demonstrator of Clinical Obstetrics in the Jefferson Medical College, Philadelphia, etc. With 150 illustrations. P. Blakiston, Son & Co., Philadelphia. Price 80c., net. 1896.

This valuable series of quiz compends is already well known to students and practitioners of medicine. This one is no departure from the standard set up by its predecessors, and contains in a terse, concise form a condensation of the best teaching of the present day in the province of gynecology. The illustrations are numerous and well executed.

---

A PRACTICAL TREATISE ON MEDICAL DIAGNOSIS. For the use of Students and Practitioners. By John H. Musser, M. D., Assistant Professor of Clinical Medicine, University of Pennsylvania, Philadelphia. New (2d) edition, thoroughly revised. In one octavo volume of 925 pages, with 177 engravings and 11 full page colored plates. Cloth, $5.00; leather, $6.00. Lea Brothers & Co., Publishers, Philadelphia and New York, 1896.

The author has dealt with this important and progressive subject in such a clear and practical manner that but a casual survey of the book will convince the reader of its value. The second edition is a complete revision of the first, and contains, in addition, numerous illustrations in black and colors. The book will be of great service to the practical worker in medicine, and to the conscientious student. A knowledge of this subject is essential to the successful practice of medicine.

